# Interactions between zinc and NRF2 in vascular redox signalling

**DOI:** 10.1042/BST20230490

**Published:** 2024-02-19

**Authors:** Fan Yang, Matthew J. Smith, Richard C.M. Siow, Dag Aarsland, Wolfgang Maret, Giovanni E. Mann

**Affiliations:** 1 School of Cardiovascular and Metabolic Medicine and Sciences, King's British Heart Foundation Centre of Research Excellence, Faculty of Life Sciences and Medicine, King's College London, 150 Stamford Street, London SE1 9NH, U.K.; 2 Department of Old Age Psychiatry, Institute of Psychiatry, Psychology and Neuroscience, King's College, London, U.K.; 3 Centre for Age-Related Medicine, Stavanger University Hospital, Stavanger, Norway; 4 Departments of Biochemistry and Nutritional Sciences, School of Life Course and Population Sciences, Faculty of Life Sciences and Medicine, King's College, London, U.K.

**Keywords:** KEAP1, NRF2, oxygen, redox signalling, zinc

## Abstract

Recent evidence highlights the importance of trace metal micronutrients such as zinc (Zn) in coronary and vascular diseases. Zn^2+^ plays a signalling role in modulating endothelial nitric oxide synthase and protects the endothelium against oxidative stress by up-regulation of glutathione synthesis. Excessive accumulation of Zn^2+^ in endothelial cells leads to apoptotic cell death resulting from dysregulation of glutathione and mitochondrial ATP synthesis, whereas zinc deficiency induces an inflammatory phenotype, associated with increased monocyte adhesion. Nuclear factor-E2-related factor 2 (NRF2) is a transcription factor known to target hundreds of different genes. Activation of NRF2 affects redox metabolism, autophagy, cell proliferation, remodelling of the extracellular matrix and wound healing. As a redox-inert metal ion, Zn has emerged as a biomarker in diagnosis and as a therapeutic approach for oxidative-related diseases due to its close link to NRF2 signalling. In non-vascular cell types, Zn has been shown to modify conformations of the NRF2 negative regulators Kelch-like ECH-associated Protein 1 (KEAP1) and glycogen synthase kinase 3β (GSK3β) and to promote degradation of BACH1, a transcriptional suppressor of select NRF2 genes. Zn can affect phosphorylation signalling, including mitogen-activated protein kinases (MAPK), phosphoinositide 3-kinases and protein kinase C, which facilitate NRF2 phosphorylation and nuclear translocation. Notably, several NRF2-targeted proteins have been suggested to modify cellular Zn concentration via Zn exporters (ZnTs) and importers (ZIPs) and the Zn buffering protein metallothionein. This review summarises the cross-talk between reactive oxygen species, Zn and NRF2 in antioxidant responses of vascular cells against oxidative stress and hypoxia/reoxygenation.

## Introduction

Oxidative stress is increasingly recognised as a contributor to redox dysregulation in various diseases such as stroke, cardiovascular diseases, diabetes and cancer [1–6]. Notably, cellular oxidative stress and increased generation of reactive oxygen species (ROS) is counter-balanced by the up-regulation of endogenous antioxidant defences. Intracellular glutathione (GSH) and antioxidant enzymes (e.g. CuZnSOD) were initially shown in the 1970s to scavenge free radicals generated during oxidative stress induced damage [7–9]. NRF2 was cloned in 1994 and described as an activator for transcriptional stimulation of β-globin genes and subsequently identified as a key transcription factor in cellular antioxidant defences [10–13], with many antioxidant genes induced by sulforaphane and other electrophilic agents [14–16]. Notably, genetic manipulation of NRF2 has identified hundreds of target genes [17], including antioxidant enzymes such as NAD(P)H:quinone oxidoreductase-1 (NQO1), heme oxygenase-1 (HO-1) and glutamate cysteine ligase (GCL) [18–20] but also regulators of protein clearance and metabolism [13,21–23].

Metals have been linked with redox signalling due to oxidative stress induced changes in intracellular labile and total metal concentrations [24,25]. As a redox-inert metal, zinc (Zn) has been implicated in protection against oxidative stress indirectly by stabilising thiol groups against free radical attack or as a promotor of ROS generation exacerbating the severity of myocardial infarction [26–29]. Thus, whether Zn is protective or promotes injury is a matter of its concentrations and the control of its cellular homeostasis. In this review, we focus primarily on the role of Zn and NRF2 interactions in redox signalling, with the aim of informing diagnostic and therapeutic strategies for cardiovascular protection in oxidative stress.

## Oxidant induced activation of the KEAP1/NRF2 transcriptional pathway

The Kelch-like ECH-associated Protein 1 (KEAP1)/NRF2 antioxidant defence pathway enables cells to counter oxidative and nitrosative stress in atherosclerosis and ischemia/reperfusion injury [30–37]. In addition to regulating cellular antioxidant defences, activation of NRF2 affects metabolism, autophagy, cell proliferation, remodelling of the extracellular matrix and wound healing [35]. Under basal conditions, cytosolic NRF2 is rapidly sequestered by KEAP1 and targeted for ubiquitination and proteasomal degradation [12,13,22,23,38]. Modification of KEAP1 cysteine residues leads to nuclear accumulation of NRF2 [12]. GSK-3β phosphorylation also affects NRF2 degradation via the adaptor protein β transducin repeats-containing proteins (β-TrCP) independent of KEAP1 and facilitates Fyn-mediated NRF2 nuclear exclusion [39,40]. In the nucleus, binding of NRF2 together with small Maf proteins to the antioxidant response element (ARE) initiates gene transcription [12,13,22].

## Zinc and cellular redox signalling

Cells utilise a diverse range of metal ions (e.g. Zn, Fe, Cu, Mn, Ca, Mg), and each cell type has a unique metal ion composition [41–44]. Zinc plays important roles in cellular metabolism and signalling, and Zn^2+^ is required as a catalytic and structural cofactor for more than 3000 human proteins [41]. Physiological concentrations of zinc have antioxidant, anti-inflammatory and anti-proliferative properties [45–50], whilst zinc deficiency or overload generates oxidative stress [51]. Intracellular zinc concentrations change dynamically to regulate rapid cellular events and slow transcriptional responses [41–43,52]. In human cells, free Zn^2+^ levels are estimated at a few hundred picomolar, yet total zinc concentration ∼100 μM [52]. Intracellular Zn^2+^ can increase transiently above low nanomolar concentrations, yet is tightly regulated by cytosolic buffering and the activity of transporters that remove Zn^2+^ ions from the cytosol [41,43,53,54]. ZnT (solute-linked carrier 30) transport proteins lower zinc concentrations through cellular efflux or uptake into cellular compartments, whilst ZIP (solute-linked carrier 39) proteins increase zinc levels through influx into cells or export from organelles [55,56]. In addition, metallothioneins (MTs), small proteins containing thiolate clusters, bind Zn^2+^ ions with a range of affinities [51,54]. Tight control of Zn^2+^ levels via multiple transporters, sequestration, redistribution, storage and elimination maintains cellular function, including thiol/disulfide redox homeostasis because the majority of intracellular zinc is bound to redox-sensitive thiols of the amino acid cysteine [52]. In this context, H_2_O_2_ or ROOH oxidation of Cys residues in proteins, in which a zinc ion is bound to a Cys residue, can lead to formation of Zn^2+^-hydroxyl species and rapid disulfide formation [57]. Moreover, several Cys residues have been identified as key for Zn-responsive transcription of MTF-1 [58].

Zinc mediated protection of the endothelium against oxidative stress [59,60] involves up-regulation of the NRF2 target GCL and increased intracellular glutathione (GSH). As Zn^2+^ plays a signalling role in endothelial nitric oxide synthase activity, maintenance of appropriate zinc levels in endothelial cells may afford further protection against oxidative stress in vascular diseases [61]. We recently reported that zinc supplementation up-regulates NRF2 targeted HO-1 protein expression and attenuates the ROS generation in human coronary smooth muscle cells [62]. Moreover, in a diabetic mouse model, zinc mediated protection against aortic oxidative damage is associated with an up-regulation of NRF2 and MT [63]. These latter studies highlight the cross-talk between zinc and NRF2 mediated redox defences.

Zinc deficiency *in vivo* is associated with impaired NRF2 regulated antioxidant defences, and notably vascular remodelling caused by a high fat diet is exacerbated by zinc deficiency and improved by zinc supplementation [64]. Excessive accumulation of Zn^2+^ in endothelial cells leads to apoptotic cell death as a consequence of dysregulation of glutathione and mitochondrial ATP synthesis [65], highlighting cross-talk between zinc, ROS and NRF2 regulated antioxidant defences [22,66–68]. Zinc deficiency induces an inflammatory endothelial cell phenotype, associated with increased monocyte adhesion which can be reversed by zinc supplementation [69]. Unlike peripheral endothelial cells, human brain endothelial cells respond to zinc depletion by tightening their barrier function [70]. As zinc transporters and MTs in human microvasculature appear to localise differentially among endothelial and smooth muscle cells [71], dietary zinc supplementation may affect endothelial and smooth muscle cells differentially.

## Interactions between zinc and NRF2 in cellular redox signalling

**Figure 1.**
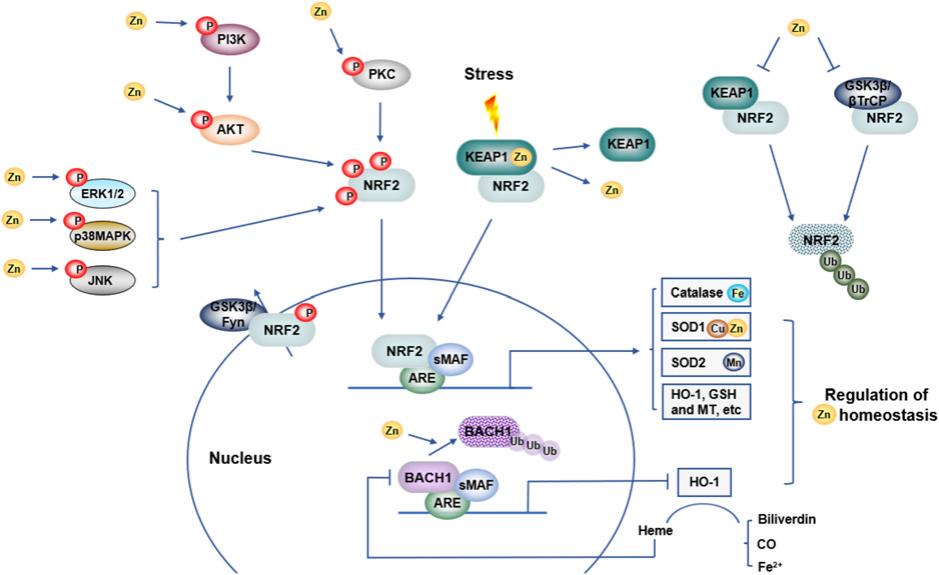
Interactions between zinc and NRF2 regulated cellular redox signalling. There are close links between zinc and NRF2. Zinc modifies KEAP1 and GSK3β, negative regulators of NRF2 signalling and promotes degradation of Bach1, a transcriptional suppressor of select NRF2 targeted genes. Zn can trigger kinases including MAPK (ERK1/2, p38MAPK, JNK), PI3K and PKC, which can modulate NRF2 phosphorylation and nuclear translocation. In turn, many proteins induced by NRF2, such as catalase, CuZnSOD, MnSOD, HO-1, and MT influence Zn homeostasis. NRF2, nuclear factor erythroid 2-related factor 2; GSH, glutathione; HO-1, heme oxygenase-1; Zn, zinc; KEAP1, Kelch-like ECH-associated protein 1; GSK3β, glycogen synthase kinase 3β; βTrCP, β Transducin repeats-containing proteins; sMaf, small Maf proteins; ARE, antioxidant response element; ZnT, zinc transporters, solute-linked carrier 30; ZIP, Zrt- and Irt-like protein, solute-linked carrier 39; MT, metallothioneins; MAPK, mitogen-activated protein kinases; PI3K, phosphoinositide 3-kinases; PKC, protein kinase C; Akt, protein kinase B; ERK1/2, extracellular signal-regulated kinase 1 and 2; BACH1, BTB and CNC homology 1.

Zinc and NRF2 independently play critical roles in redox signalling, whilst they also closely regulate each other (Figure 1). Two main negative regulators of NRF2, KEAP1 and GSK-3β, can be modified by Zn and thereby lose their function in mediating NRF2 ubiquitination and degradation. KEAP1, acting as an electrophile sensor and Zn-binding factor, can release Zn from cysteine 273 and 288 under oxidative stress [12,72,73].

Numerous studies have shown that NRF2 is phosphorylated by kinases, including mitogen-activated protein kinases (MAPK), phosphoinositide 3-kinases (PI3K) and protein kinase C (PKC), promoting NRF2 activity and accumulation in the nucleus [74–79]. Activation of NRF2 in cultured rat cardiomyocytes affords protection against redox stress involving PI3K/protein kinase B (Akt) and extracellular signal-regulated kinase 1 and 2 (ERK1/2) pathways [80]. Notably, Zn supplementation of human renal tubular (HK11) cells leads to increased Akt and GSK-3β phosphorylation and a decrease in the NRF2 nuclear exporter Fyn [81]. Zn can trigger the activity of ERK1/2, inducing NRF2 dissociation from KEAP1 and NRF2 translocation into the nucleus, leading to an up-regulation of antioxidant genes such as HO-1 and NQO1 [78,82–85].

The transcription factor BTB and CNC homology 1 (BACH1) is expressed in mammalian tissues and functions primarily as a transcriptional suppressor of select NRF2 targeted genes (e.g. HO-1 and NQO1), whilst up-regulation of GSH-related genes (e.g. GCLM and GCLC) appears to be independent of BACH1 [14,86]. In turn, BACH1 is a NRF2 targeted gene and has a functional Maf recognition element site near the transcription start site of BACH1 transcript variant 2, which can be up-regulated by NRF2 overexpression and NRF2 activators in human umbilical vein endothelial cells and human HepG2 cells [87,88]. HO-1 catalyses the first and rate-limiting step of heme degradation into biliverdin, CO and free iron [19,89]. In the presence of lower concentrations of heme within a cell, BACH1 binds to the ARE together with small Maf protein, whilst in the presence of higher concentrations of heme, BACH1 is inactivated by binding to heme, leaving ARE sites available to NRF2 binding [90]. In this context, Ogawa et al. [91] reported that heme inhibits the repressor BACH1 in human embryonic kidney HEK293 cells, abrogating its repression of HO-1. Further studies are required to establish such regulation involving heme, HO-1, Bach1 and NRF2 in vascular cell types. As Zn mediates ubiquitination and degradation of BACH1, this would lead to an increased capacity of NRF2 to up-regulate target antioxidant genes [68,92].

## Influence of oxidative stress and hypoxia/reoxygenation

Dysregulation of Zn homeostasis is associated with ischemia/reperfusion injury [93–97]. Serum zinc levels are low in patients undergoing cardiac surgery and an association between zinc deficiency and postoperative outcomes remains to be established, although supplemental zinc has beneficial effects in recovery post-surgery [98]. Ischemia/reperfusion is characterised by increased generation of intracellular ROS that are causally linked to elevated Zn^2+^ levels [28,94,99]. Increases in intracellular Zn^2+^ by thiol-reactive oxidants have been implicated in altered excitation-contraction coupling in cardiomyocytes [100–102]. Total zinc levels in cardiomyocytes exposed to hypoxia may be elevated due to increased expression of zinc importers (ZIPs), with impaired accumulation of intracellular zinc during reoxygenation due to overexpression of specific zinc transporters and down-regulation of ZIP expression [103]. More recent studies in cardiomyocytes have shown that intermittent hypoxia affords protection against hypoxia/reoxygenation by limiting cytosolic zinc overload [104], which like Ca^2+^ is a trigger of cell death.

Oxidative stress and/or hypoxia release zinc from MT, a key metal ion-binding protein in cells [51]. Metal response element binding transcription factor-1 (MTF-1), a zinc finger transcription factor, plays a key role in detoxifying heavy metals and protecting cells against hypoxia and oxidative stress [105]. Activation of MTF-1 leads to increased expression of MT and zinc transporter 1 (ZnT1), and sequestration and extrusion of transiently elevated labile Zn^2+^ [105,106]. Notably, transcriptional activation of MTF-1 and NRF2 by oxidants is linked via a pool of available zinc controlled by MTF-1 [51], and both transcription factors are involved in the protection against hypoxia and oxidative stress [105].

We recently investigated effects of NRF2 signalling on zinc content and expression of ZnT1 and MT1 in human coronary artery endothelial and smooth muscle cells transfected with NRF2-siRNA or a NRF2 overexpression vector. Using ICP-MS analysis, we established that NRF2 activation with sulforaphane or NRF2 overexpression tended to increase total zinc content in coronary endothelial cells with negligible changes detected in coronary smooth muscle cells [107]. Furthermore, in coronary endothelial cells, adapted long-term (5 days) to physiological oxygen levels (5 kPa), NRF2 overexpression also increases ZnT1 but not MT1 expression [107]. In HepG2 cells, NRF2 activation by tert-butylhydroquinone is associated with significant increases in ZnT1, ZnT3, and ZnT6 mRNAs levels and decreases in ZnT10 mRNA levels [66], though this study did not provide evidence for NRF2 directly regulating zinc transporters.

Previous studies in coronary artery endothelial cells reported that ZnT1, ZnT2 and MT1 expression are significantly decreased by low or elevated zinc, while zinc depletion up-regulates ZIP2 and ZIP12 expression [108]. In umbilical vein endothelial cells, low or oscillatory shear stress increases ZnT1 and MT expression and lowers intracellular zinc [109]. As oscillatory shear is associated with diminished NRF2 signalling [110] and decreased nitric oxide generation [111] in atheroprone regions of the endothelium, this together with increased ZnT1 and MT expression may explain decreased intracellular zinc levels [109].

## Importance of physiological oxygen levels for assessing interactions between zinc and NRF2 in culture

In view of its antioxidant, anti-inflammatory and anti-proliferative actions, it is worth noting that the mechanisms underlying zinc mediated protection against ischemia/reoxygenation have been studied mainly in cells cultured under atmospheric oxygen levels. Although Zn supplementation enhances NRF2 nuclear accumulation in coronary artery smooth muscle cells adapted to either atmospheric oxygen (18 kPa) or physiological normoxia (5 kPa), Zn induced up-regulation of NRF2 targeted HO-1/NQO1 expression is markedly attenuated in cells under 5 kPa O_2_ [62]. Under these experimental conditions, Zn supplementation only protected coronary artery smooth muscle cells against reoxygenation induced free radical generation during culture under 18 kPa O_2_ [62], confirming similar findings for reoxygenation induced superoxide generation in brain microvascular endothelial cells [15]. The diminished ability of Zn to protect against reoxygenation induced free radical damage in smooth muscle cells may reflect a lower redox stress experienced by cells under physiological normoxia [6,112] and/or changes in the expression/activity of zinc importers and exporters and MTs.

## Conclusions and future research

Oxidative stress affects cell proliferation, migration, differentiation, redox phenotype as well as metal profiles in a cell-specific manner [6,15,62,107]. Recent evidence suggests that a zinc-dependent aminopeptidase, dipeptidylaminopeptidase III (DPP3), interacts with KEAP1, leading to NRF2 nuclear translocation and gene transcription [113]. In the context of oxidative stress, H_2_O_2_ strongly enhances the interaction between DPP3 and KEAP1, potentially contributing to NRF2 mediated protection against ischemia/reperfusion injury [114].

As pericellular oxygen levels affect the redox phenotype of vascular cells [14,15,62,107,115,116] and total intracellular zinc content [107] during culture *in vitro*, we encourage researchers to correlate changes in labile and total metal content, especially Zn, with redox signalling in cell models adapted long-term (5 days) to physiological oxygen levels which vary in different cell types and tissues. This has important implications for identifying potential biomarkers of oxidative-related diseases, with critical consequences for disease diagnosis and prognosis. Such studies may create opportunities for new clinical tools relating perturbations in zinc with disease progression, and the design of drugs for restoration of metal homeostasis, with implications for the heart, brain and wider field of vascular biology.

## Perspectives

- Zn supplementation affords antioxidant protection via complex interactions with NRF2 and MTF-1 signalling pathways.
- Zn activates NRF2 target genes by modifying KEAP1 and GSK3β and interacts with multiple other signalling pathways.
- Investigating molecular interactions between zinc, NRF2 and MTF-1 under physiological oxygen *in vitro* may inform the design of drugs and nutritional interventions to restore metal homeostasis in vascular and other diseases associated with redox distress.

## Abbreviations

Akt: protein kinase B
ARE: antioxidant response element
βTrCP: β Transducin repeats-containing proteins
DPP3: dipeptidylaminopeptidase III
ERK1/2: extracellular signal-regulated kinase 1 and 2
GCL: glutamate cysteine ligase
GSH: glutathione
GSK3β: glycogen synthase kinase 3β
HO-1: heme oxygenase-1
KEAP1: Kelch-like ECH-associated protein 1
MAPK: mitogen-activated protein kinases
MT: metallothionein
MTF-1: metal regulatory transcription factor 1
NQO1: NAD(P)H:quinone oxidoreductase-1
NRF2: nuclear factor erythroid 2-related factor 2
PI3K: phosphoinositide 3-kinases
PKC: protein kinase C
ROS: reactive oxygen species
sMaf: small Maf proteins
Zn: zinc
